# Married

**Published:** 1875-04

**Authors:** 


Married in Rome, Georgia, on the 10th of March, John B. King, of Savannah, Georgia, to Mary, daughter of Dr. Robert Battey, of Atlanta, Georgia.

